# Effect of Chlorine Exposure on the Survival and Antibiotic Gene Expression of Multidrug Resistant *Acinetobacter baumannii* in Water 

**DOI:** 10.3390/ijerph110201844

**Published:** 2014-02-07

**Authors:** Deepti Prasad Karumathil, Hsin-Bai Yin, Anup Kollanoor-Johny, Kumar Venkitanarayanan

**Affiliations:** 1 Department of Animal Science, 3636 Horsebarn Hill Rd Ext., Unit 4040, University of Connecticut, Storrs, Connecticut, CT 06269, USA; E-Mails: deepti.karumathil@uconn.edu (D.P.K.); hsin-bai.yin@uconn.edu (H.B.Y.); 2 Department of Animal Science, University of Minnesota, St. Paul, Minnesota, MN 55108, USA; E-Mail: anupjohn@umn.edu

**Keywords:** *Acinetobacter baumannii*, water, chlorine, disinfection, antibiotic resistance

## Abstract

*Acinetobacter baumannii* is a multidrug resistant pathogen capable of causing a wide spectrum of clinical conditions in humans. *Acinetobacter* spp. is ubiquitously found in different water sources. Chlorine being the most commonly used disinfectant in water, the study investigated the effect of chlorine on the survival of *A. baumannii* in water and transcription of genes conferring antibiotic resistance. Eight clinical isolates of *A. baumannii*, including a fatal meningitis isolate (ATCC 17978) (~10^8^ CFU/mL) were separately exposed to free chlorine concentrations (0.2, 1, 2, 3 and 4 ppm) with a contact time of 30, 60, 90 and 120 second. The surviving pathogen counts at each specified contact time were determined using broth dilution assay. In addition, real-time quantitative PCR (RT-qPCR) analysis of the antibiotic resistance genes (efflux pump genes and those encoding resistance to specific antibiotics) of three selected *A. baumannii* strains following exposure to chlorine was performed. Results revealed that all eight *A. baumannii* isolates survived the tested chlorine levels during all exposure times (*p* > 0.05). Additionally, there was an up-regulation of all or some of the antibiotic resistance genes in *A. baumannii*, indicating a chlorine-associated induction of antibiotic resistance in the pathogen.

## 1. Introduction

Multidrug resistant (MDR) *Acinetobacter baumannii* is a major hospital-borne pathogen causing a wide spectrum of clinical conditions with significant mortality rates [1,2,3,4,5,6]. *A. baumannii* strains are equipped with a multitude of antibiotic resistance mechanisms rendering them resistant to most of the currently available antibiotics [1,3]. *A. baumannii* has a remarkable ability to persist for prolonged periods of time in the hospital environment in biofilms, thereby insulating it from disinfectants, and serving as a continuous source of infection [7,8,9,10,11]. In health-care environments, a variety of surfaces, including tabletops, bed rails, sinks, door handles, floors, mattresses, and pillows have been implicated as potential sources of *A. baumannii* [3,12].

Water and soil are considered as a major habitat for *A. baumannii* although the pathogen has been isolated from other sources, including foods, arthropods, animals, and humans [13,14,15,16,17]. Moreover, protozoans such as *Acanthamoeba* have been reported to support the growth of *A. baumannii*, and act as its reservoir in water [18]. Recently, studies indicating the potential presence of *A. baumannii* in water systems have been reported from multiple parts of the world [19,20]. The ability of *A. baumannii* to thrive in water may result in fatal infections in all age groups [21].

Chlorine has long been used as a disinfectant in drinking water and in swimming pools to inactivate pathogenic microorganisms, thereby making water safe for human use [22,23,24]. In the United States, the Environmental Protection Agency (EPA) recommends a maximum free chlorine level of 4 ppm in drinking water [25]. In addition to the standards described for chlorine in drinking water, the Centers for Disease Control and Prevention (CDC) have recommended 1–3 ppm free chlorine in swimming pool water for recreational purposes [26]. However, a variety of microorganisms have been recovered from drinking water distribution systems that maintained chlorine levels between 0.5–1.0 ppm, indicating that low levels of chlorine may not inactivate harmful microorganisms [27,28,29]. It is also reported that chlorine used in potable water and sewage can selectively promote the survival of antibiotic resistant bacteria [23,30,31,32]. For instance, drinking water with suboptimal levels of chlorine selectively supported the survival of multidrug resistant *Pseudomonas aeruginosa* [23]. 

Since *A. baumannii* could potentially contaminate drinking or recreational water, and chlorine at the recommended levels may not be effective in killing the pathogen, the current research investigated the viability of *A. baumannii* in water containing chlorine at the recommended levels for potable and recreational usage. In addition, the effect of chlorine on various antibiotic resistance genes in *A. baumannii* was investigated.

## 2. Experimental Section

### 2.1. A. baumannii Strains and Growth Conditions

Eight strains of *A. baumannii*, including 251847, 134882 (wound infection), 173795 (wound infection), 474030 (blood), 190451 (respiratory tract), 163731 (respiratory tract), and 251352 (source unknown) kindly gifted by the International Health Management Associates (IHMA, Schaumburg, IL, USA), and ATCC 17978 (brain) were used in the chlorine survival study. The antibiotic resistance profile of the eight clinical strains was amikacin (MIC 64 µg/mL), amoxicillin (MIC 32 µg/mL), cefepime (MIC 32 µg/mL), ceftazidime (MIC 32 µg/mL), ceftriaxone (MIC 64 µg/mL), imipenem (MIC 4–32 µg/mL), levofloxacin (MIC 8 µg/mL), meropenem (MIC 16 µg/mL), minocycline (MIC 1–16 µg/mL), and piperacillin (MIC 128 µg/mL). Each strain of *A. baumannii* was grown individually on MDR *Acinetobacter*, and Leeds *Acinetobacter* agars (Hardy Diagnostics, Santa Maria, CA, USA), and an individual colony from these media was sub cultured at least 3 times in tryptic soy broth (TSB; Difco, Sparks, MD, USA ) for 24 h at 37 °C with shaking (200 rpm). After the subcultures, the bacterial cells were harvested from an overnight culture by centrifugation at 3,600 × g for 30 min at 4 °C. The cells were washed twice in sterile phosphate buffered saline (PBS, pH = 7.2), and the bacterial cell pellet was finally resuspended in PBS to get a final concentration of 10^9^ CFU/mL. The bacterial population in the inoculum was confirmed by broth dilution and surface plating on tryptic soy agar (TSA; Difco) plates.

### 2.2. A. baumannii Survival Assay

The effect of chlorine on *A. baumannii* viability in water was determined using a published protocol [33]. Deionized, non-chlorinated (EMD Millipore, Billerica, MA, USA) water was used for the study. For each experiment, different chlorine concentrations (0.2, 1, 2, 3 and 4 ppm) in water were achieved by adding a standard chlorine solution (Aqua Solutions, Deer Park, TX, USA) to pre-sterilized deionized water. The final concentration of free chlorine in water was confirmed using a digital titrator (Pocket colorimeter^TM^II, Hach, Loveland, CO, USA). One mL of *A. baumannii* suspension containing 10^9^ CFU/mL was added to 99 mL of the sterile deionized water containing chlorine at the specified concentrations in a 200 mL Erlenmeyer flask. After thorough mixing, 1 mL samples were taken at 30, 60, 90 and 120 s, and transferred to 9.0 mL neutralizing broth for buffering chlorine (NB, Difco). Serial ten-fold dilutions in PBS were made and 0.1 mL of each dilution was surface plated on duplicate on TSA plates. The plates were incubated at 37 °C for 24 h. After enumeration of the colonies, the counts were expressed as log_10_ CFU/mL. The colonies on TSA were confirmed as *A. baumannii* by streaking on MDR and Leeds agar plates. Duplicate samples were included for each treatment and control, and the experiment was replicated three times. 

### 2.3. Antibiotic Resistance Gene Expression

#### 2.3.1. RNA Isolation and cDNA Synthesis

Three selected strains of *A. baumannii* (ATCC 17978, 474030, 251847) were grown on MDR plates and sub cultured in TSB separately as before. The bacterial populations in the cultures were confirmed to contain ~8 log_10_ CFU/mL by plating appropriate dilutions on TSA. The overnight culture from this tube was centrifuged, washed twice, and reconstituted in PBS as described before. One mL of each of this reconstituted culture was transferred to tubes containing 9 mL of sterile deionized water containing 2 ppm of free chlorine. Tubes with no added chlorine served as control. The 2-ppm concentration was chosen for the RT-qPCR analysis since the United States Department of Health (USDH) recommends the free chlorine concentration range of 1 to 3 ppm to disinfect swimming pools [26]. Both set of tubes were incubated at 25 °C for 15 min. The bacterial culture from each tube was centrifuged at 12,000 × g for 2 min at 4 °C. The supernatant was discarded and the pellet was added with 0.5 mL of sterile water and 1 mL of RNA protect reagent (Qiagen, Valencia, CA, USA). The mixture was then incubated at 25 °C for 5 min. The RNeasy mini kit (Qiagen) was used for extracting total RNA from the control and chlorine-treated samples. The RNA was quantified using NanoDrop (ThermoFisher Scientific, Waltham, MA, USA) by measuring the absorbance at 260 and 280 nm. Super-script II reverse transcriptase kit (Invitrogen, Carlsbad, CA, USA) was used for cDNA synthesis from the extracted RNA.

#### 2.3.2. Real-Time Quantitative PCR (RT-qPCR)

The following *A. baumannii* genes were studied in the gene expression analysis: efflux pump genes *adeA*, *adeB*, *adeC*, and *abeM*; chloramphenicol resistance gene, *cmr*, β-lactam resistance gene, *blaP*; sulphonamide resistance gene *sul1*; tetracycline resistance gene, *tetA*, and multidrug resistance protein B, *mdrp*. 

The primers specific for the genes and for the endogenous control (16S rRNA) were designed using the Primer Express software ^®^ (Applied Biosystems, Foster City, CA, USA) based on *Acinetobacter baumannii* AB0057 genome (CP001182.1) published in the NCBI database [34]. Custom synthesized primers for each gene were obtained from Integrated DNA Technologies (Foster City, CA, USA). The primers used in the study and their parent gene function are provided in Table 1. 

**Table 1:** Primers used in the study.

Gene	Sequence (5’→3’)	Function
*adeA* (F)	TGACCGACCAATGCACCTT	Efflux pump
(R)	GCAACAGTTCGAGCGCCTAT	
*adeB* (F)	CCGATGACGTATCGAAGTTAGGA	Efflux pump
(R)	CCGATGACGTATCGAAGTTAGGA	
*adeC* (F)	ACGGCCCCAGAAGTCTAGTTC	Efflux pump
(R)	CGATTAACCCCAATAACCCAGTT	
*adeM* (F)	GGTACATGGAAGCCCAGTTCTT	Efflux pump
(R)	CCACTTTCTCTTGCCATTGCT	
*blaP* (F)	ACACTAGGAGAAGCCATGAAGCTT	Beta-lactam resistanceAntibiotics
(R)	GCATGAGATCAAGACCGATACG	
*cmr* (F)	CTATTTGAATTTGCGGTTTATATTGG	Chloramphenicol resistance
(R)	TGCACTTACACCGAAATCTTCAG	
*ami* (F)	TGATCCCGTAAATGAGTTGAATTG	Aminoglycoside resistance
(R)	GCGGGCAAATGTGATGGTA	
*sul1* (F)	GGCATGACAATAGGGCAGTTG	Sulphonamide resistance
(R)	CCAAAAAGTAGATGATAATACCGGTAAA	
*tetA* (F)	CTGCGCGATCTGGTTCACT	Tetracycline resistance
(R)	GCATACAGCGCCAGCAGAA	
*mdrp* (F)	GTACGGCTTCTAGACCCACCATTTT	Multiple drug resistanceprotein
(R)	ACAAAGAGCCGTGCACAGTTT	
rRNA-16S(F)	TCGCTAGTAATCGCGGATCACGCTGGCGGC	Endogenous control
rRNA-16S(R)	GACGGGCGGTGTGTACAAG	

Note: (F), forward; (R), reverse.

RT-qPCR was done with the ABI Prism 7900 sequence detection system (Applied Biosystems) using the SYBR green assay under custom thermal cycling conditions with the normalized cDNA as template [35]. The samples were analyzed in duplicates and standardized against 16S rRNA gene expression. The relative changes in mRNA expression levels were determined using comparative threshold cycle (CT) method (2^−^^ΔΔCT^) between the chlorine-exposed and chlorine non-exposed *A. baumannii.*

### 2.4. Statistical Analysis

The counts of *A. baumannii* in the control and treated samples were logarithmically transformed (log_10_ CFU/mL) to aid in statistical analysis. Since there was no significant difference in bacterial counts between the strains following exposure to chlorine treatment, the data from the eight strains were pooled and averaged. Data analysis was done using the PROC-MIXED procedure of statistical analysis software (SAS version 9.2; SAS Institute Inc., Cary, NC, USA). Fisher’s least significance test (LSD) was used to determine the differences between the means at a *p* level of ≤0.05.

## 3. Results and Discussion

### 3.1. Effect of Chlorine on A. baumannii Survival

In order to determine if *A. baumannii* survived the recommended levels of chlorine, we determined the survival of the pathogen exposed to 0 to 4 ppm of free chlorine for 30, 60, 90 and 120 seconds in deionized water. All eight *A. baumannii* controls where no chlorine was added yielded 10^7^ CFU/mL bacteria at all the time points tested. Table 2 shows the results of *A. baumannii* counts as a mean of log CFU/mL and standard deviation of all the eight strains. When exposed to free chlorine levels ranging from 0.2 to 4 ppm, all the eight strains of *A. baumannii* survived with no significant decrease in their counts throughout the sampling period (*p* > 0.05). 

Chlorine is generally used to disinfect both potable and recreational water with stipulated standards for inclusion as determined by the EPA and CDC. However, previous studies have indicated that chlorine was not completely effective in inactivating several pathogenic bacteria. For example, *Yersinian enterocolitica*, *Yersinia pestis*, *Pasteurells multocida* and *Hafnia alvei* were isolated from chlorine treated sewage water, indicating the inefficiency of chlorine in killing these pathogens [32]. In another study [33], an isolate of *E.coli* O157:H7 with tolerance of up to 2 ppm free chlorine treatment after one minute exposure, among six other isolates were tested in their study. In yet another study, *Escherichia coli* isolated from a chlorine-treated swimming pool were found to be resistant to chlorine for up to nine passages [36]. High tolerance of bacteria to disinfectants could either be intrinsic or resulting from mutation [37]. Additionally, wide spread use of disinfectants has been reported to trigger the selection of resistant strains [37].

**Table 2:** Effect of different concentrations of chlorine on the survival of *A. baumannii* in deionized water *.

Free Chlorine (ppm)	*A. baumannii* Counts (mean ± SD **)			
	30 seconds	60 seconds	90 seconds	120 seconds
0	7.34 ± 0.24	7.38 ± 0.23	7.37 ± 0.26	7.35 ± 0.23
0.2	7.37 ± 0.15	7.39 ± 0.18	7.37 ± 0.18	7.39 ± 0.20
1	7.35 ± 0.18	7.34 ± 0.18	7.33 ± 0.20	7.34 ± 0.22
2	7.27 ± 0.51	7.27 ± 0.53	7.25 ± 0.50	7.20 ± 0.55
3	7.28 ± 0.48	7.29 ± 0.47	7.26 ± 0.46	7.24 ± 0.50
4	7.28 ± 0.46	7.29 ± 0.50	7.25 ± 0.47	7.24 ± 0.49

Note: * Non significant at *p* > 0.05, ** mean and SD of all the eight strains of *A. baumannii*.

### 3.2. Effect of Chlorine on A. baumannii Antibiotic Resistance Genes

Since we observed that *A. baumannii* could survive all the tested concentrations of chlorine in water, we investigated the effect of chlorine exposure on major antibiotic resistance determinants using RT-qPCR. The effect of chlorine on the expression of ten major antibiotic resistance genes conferring resistance to multiple antibiotics in *A. baumannii* was studied in ATCC strain 17978, 251847, and 474030. 

**Figure 1:**
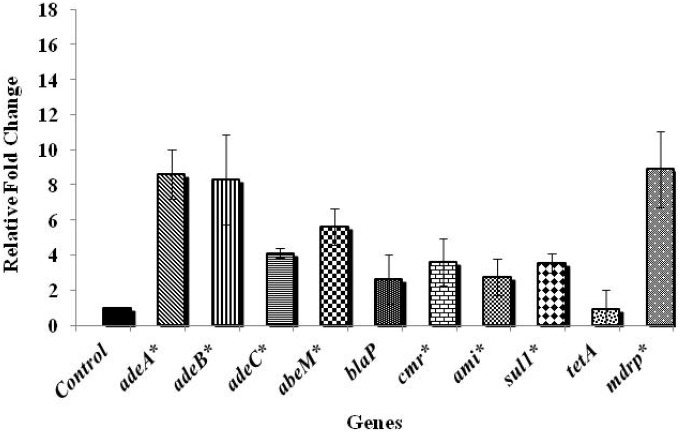
Effect of chlorine exposure on antibiotic resistance gene expression in *A. baumannii* ATCC 17978.

Note: * Genes significantly different from the control at *p* < 0.05.

The ATCC strain was selected for the gene expression analysis since it has been widely studied, and was isolated from a 4-month-old infant who died of fatal meningitis resulting from an acute infection. The results on the effect of chlorine exposure on antibiotic resistance genes in the ATCC strain are shown in Figure 1.

**Figure 2:**
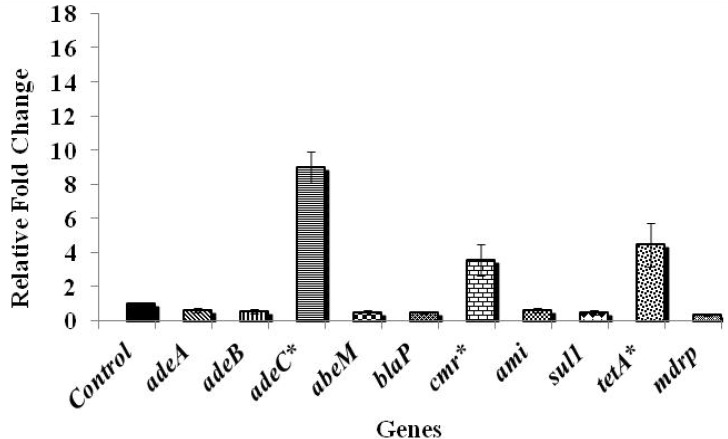
Effect of chlorine exposure on antibiotic resistance gene expression in *A. baumannii* 251847.

Note: * Genes significantly different from the control at *p* < 0.05.

**Figure 3:**
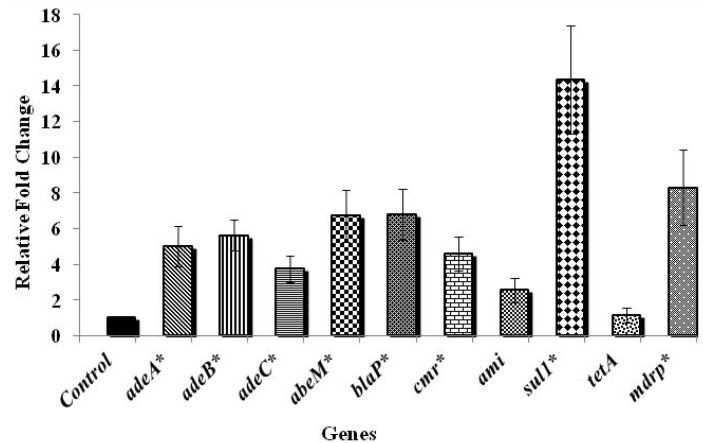
Effect of chlorine exposure on antibiotic resistance gene expression in *A. baumannii* 474030.

Note: * Genes significantly different from the control at *p* < 0.05.

Among the various genes tested, those controlling antibiotic efflux pumps in *A. baumannii*, namely *adeA*, *adeB*, and *abeM* were significantly up-regulated by more than six fold when compared to the control, while the efflux pump gene, *adeC* and the gene encoding chloramphenicol resistance, *cmr* were up-regulated by about four folds (*p* < 0.05). A three-fold up-regulation was noticed in *blaP* that confers resistance to β-lactam group of antibiotics upon exposure to chlorine (*p* > 0.05). Exposure to chlorine also resulted in an increase in the expression of aminoglycoside (*ami*) and sulphonamide (*sul1*) resistance genes by three- and four-folds, respectively (*p* < 0.05). In addition, an eight-fold up-regulation (*p* < 0.05) of the gene encoding multiple drug resistance protein (*mdrp*) in *A. baumannii* was observed following exposure to chlorine. However, no significant change in the expression of tetracycline resistance encoding gene, *tetA* was observed upon exposure to chlorine (*p* > 0.05). The results revealed that the efflux pump genes were more up-regulated compared to the antibiotic resistance genes (Figure 1), highlighting the involvement of efflux pump mechanisms on exposure to chlorine. Figure 2 shows the antibiotic gene expression in *A. baumannii* 251847, where a significant up-regulation was observed in the expression of *adeC*, *cmr* and *tetA* by nine, four- and five-folds, respectively (*p* < 0.05). However, the expression of all the other genes tested was decreased following chlorine exposure (*p* < 0.05). In *A. baumannii* 474030, a significant up-expression of all genes except *tetA* (Figure 3) was observed after exposure to chlorine (*p* < 0.05), which was similar to the trend observed in the ATCC isolate. Although the reason behind the varied response in gene expression in different *A. baumannii* isolates is not known, an earlier study reported that *A. baumannii* strains show a difference in their susceptibility towards the same antibiotics [38]. To summarize, despite variations in the expression levels of specific genes in the three isolates, exposure to chlorine induced the expression of multiple antibiotic resistance genes in all the three isolates of *A. baumannii* studied.

Although preventative chlorine levels are in use, several reports suggest that chlorine at suboptimal levels could induce the expression of critical genes in pathogenic bacteria. Shi and coworkers reported that chlorine enhanced the expression of antibiotic resistance genes in diverse microbial populations isolated from drinking water [39]. Similarly, an increase in the expression of several antibiotic resistance genes was noted in *E.coli* and *P. aeruginosa* by other research groups [23,40]. It was observed that exposure to chlorine, induced a stress tolerance in bacteria making them more resistant to antibiotics [30,31]. It was also reported that chlorine exposure could induce over expression of efflux pumps resulting in the pumping out of disinfectants and antibiotics by bacteria [41]. 

## 4. Conclusions

The current investigation indicated that a free chlorine concentration of up to 4 ppm was not effective in killing multidrug resistant *A. baumannii* isolates. All the *A. baumannii* isolates were able to survive the recommended levels of chlorine in water. Further, chlorine exposure was found to increase the expression of efflux pumps and genes conferring resistance to chloramphenicol, sulphonamides, and beta-lactam group of antibiotics in *A. baumannii*. These observations indicate the inefficiency of currently used chlorine concentrations in killing *A. baumannii* in water, thereby warranting additional research and corrective measures. In addition, further studies are required to understand the mechanism behind chlorine-induced gene expression in *A. baumannii*, and its significance to public health.
